# Bioinformatic Identification of Peptidomimetic-Based Inhibitors against *Plasmodium falciparum* Antigen AMA1

**DOI:** 10.1155/2014/642391

**Published:** 2014-12-18

**Authors:** Asrar Alam

**Affiliations:** Department of Biological Sciences, Tata Institute of Fundamental Research, Homi Bhabha Road, Colaba, Mumbai 400005, India

## Abstract

*Plasmodium falciparum* apical membrane antigen 1 (PfAMA1) is a valuable vaccine candidate and exported on the merozoite surface at the time of erythrocyte invasion. PfAMA1 interacts with rhoptry neck protein PfRON2, a component of the rhoptry protein complex, which forms the tight junction at the time of invasion. Phage display studies have identified a 15-residue (F1) and a 20-residue (R1) peptide that bind to PfAMA1 and block the invasion of erythrocytes. Cocrystal structures of central region of PfAMA1 containing disulfide-linked clusters (domains I and II) with R1 peptide and a peptide derived from PfRON2 showed strong structural similarity in binding. The peptides bound to a hydrophobic groove surrounded by domain I and II loops. In this study, peptidomimetics based on the crucial PfAMA1-binding residues of PfRON2 peptide have been identified. Top 5 peptidomimetics when checked for their docking on the region of PfAMA1 encompassing the hydrophobic groove were found to dock on the groove. Drug-like molecules having structural similarity to the top 5 peptidomimetics were identified based on their binding ability to PfAMA1 hydrophobic groove in blind docking. These inhibitors provide potential lead compounds, which could be used in the development of antimalarials targeting PfAMA1.

## 1. Introduction

*Plasmodium falciparum* apical membrane antigen 1 (PfAMA1) is a low abundance integral membrane protein located at the apical region of merozoites at late asexual blood stages [1]. AMA1 is a protective antigen against experimental malaria. Native and recombinant AMA1 have shown protection in animal malaria models [2–6]. Anti-AMA1 antibodies are inhibitory to parasite invasion [6].* In vitro* host cell invasion inhibition by anti-AMA1 antibodies could also be strain-specific due to sequence polymorphism [7–9]. Anti-AMA1 antibodies have been detected in individuals from malaria endemic regions and affinity-purified antibodies from these individuals have shown* in vitro* inhibitory activity [10]. AMA1 is expressed in two life cycle stages of the parasite, sporozoites and blood stage merozoites [11], making it an ideal candidate for a non-stage-specific vaccine. AMA1 is essential for the maintenance of blood stages of the parasite as attempts to knockout PfAMA1 gene have not been successful [12]. Full-length AMA1 is an 83-kDa polypeptide that is translocated to the apical organelle microneme at the time of invasion [13, 14], when the 83-kDa polypeptide undergoes N-terminal processing to form a 66-kDa form that is translocated to the merozoite surface [1, 15].

Structurally, AMA1 is a type I integral membrane protein, consisting of an ectoplasmic domain, a transmembrane domain, and a C-terminal cytoplasmic domain [16]. Sixteen cysteine residues are encoded in all characterized* Plasmodium* AMA1 proteins, which can be categorized into three domains based on disulfide-bonding pattern [17, 18]. Correct disulfide bonding was found to be essential for inducing protective immune response against AMA1 [3]. Crystal structure of ectoplasmic domain of* Plasmodium vivax* AMA1 and mapping of invasion inhibitory antibodies against PfAMA1 revealed that domain II is important for its biological functions. Invasion inhibitory monoclonal antibody 4G2 recognized a conformational epitope, which required both domains I and II [19]. Domains I and III are both targets of inhibitory antibodies and clustering of polymorphism around them suggests that both are targets for protective immune response in humans [18, 20–22]. Although AMA1 is a highly polymorphic protein, the central two-third region (domains I and II) is relatively conserved between* Plasmodium* and other apicomplexan parasites, whereas domain III is not well conserved. The central region (domains I and II) consists of two PAN or apple domains. The crystal structure of the central region has revealed the presence of polymorphic residues on one face with most highly polymorphic residues surrounding a hydrophobic groove [23].

In an attempt to identify peptides that bind to PfAMA1 and block its function, a random phage display library of 15-residue peptides was panned on recombinant protein. This screening led to identification of three peptides (F1, F2, and F3) binding to a similar region of the protein. F1 (GWRLLGFGPASSFM) had the highest binding affinity. Alanine scanning mutagenesis identified that resides 5 to 9 (LGFGP) of F1 were crucial for binding and N- and C-terminal residues were not essential. F1 also inhibited merozoite invasion of human red blood cells (RBCs)* in vitro* [24]. Solution state structures of synthetic F1 and F2 peptides were analyzed by NMR. F1 peptide contained a *β*-turn in the crucial region for binding identified by alanine scan. A C-terminal truncated version of F1 and a disulfide bonded peptide with similar sequence were able to bind to PfAMA1 as they contained type I *β*-turn structural motif but a partially scrambled peptide lacking this structural motif was unable to bind. F2 peptide was unstructured in solution and bound weakly. Weak binding of F2 as compared to F1 was suggestive of secondary structural requirements for binding [25]. In another study, using phage display screen to identify peptides binding to recombinant PfAMA1, a 20-mer peptide (R1) was identified that bound to PfAMA1 and inhibited merozoite invasion* in vitro*. This peptide was able to compete in binding of F1 peptide to PfAMA1 and was at least 5-fold more effective in invasion inhibition. Solution state NMR structure of the peptide was determined revealing the presence of two structured regions encompassing residues 5 to 10 and 13 to 17, both involving turn conformations [26]. In the present study, molecular docking of F1 peptide on PfAMA1 structure, and its comparison to other peptide-AMA1 cocrystal structures, has been carried out.

In* Toxoplasma gondii*, rhoptry neck protein 2 (RON2) is the receptor for AMA1 [27]. RON2 is a transmembrane component of the RON protein complex that is secreted into the host cell during invasion and subsequently integrated into the cell membrane [27, 28]. The interaction between AMA1 and RON2 was also confirmed in* P. falciparum* [29, 30]. Richard et al. (2010) demonstrated that the peptide R1 was able to block interaction between AMA1 and the RON complex in* P. falciparum*, although the effectiveness of this peptide was limited to a subset of parasite isolates due to AMA1 polymorphism. In the presence of the peptide, merozoites were able to make apical contact with RBCs but the formation of a functional moving junction could not ensue [31].

The cocrystal structures of PfAMA1 ectodomains (DI and DII) in complex with a peptide derived from PfRON2 extracellular domain PfRON2sp1 (residues 2021–2059) and its truncated version PfRON2sp2 (residues 2027–2055) revealed that both the peptides are overlaid on the same region of the protein. By surface plasmon resonance, binding affinity of PfRON2sp1 was found to be 25-fold higher than PfRON2sp2; hence PfRON2sp1 was chosen for further analysis. The cocrystal structure of R1 peptide with PfAMA1 ectodomains was also determined revealing that two R1 molecules bound to one molecule of PfAMA1, denoted as R1-major and R1-minor. R1-major bound deeply in the hydrophobic groove whereas R1-minor bound above R1-major but made fewer contacts [32].

In PfAMA1-PfRON2 interaction, receptor-binding site of PfAMA1 comprises the hydrophobic groove and a region that becomes exposed by displacement of the flexible domain II loop upon binding. Comparison of cocrystal structures PfRON2sp1 and R1 peptides bound to PfAMA1 exhibited strong structural similarity. Mutagenesis studies identified key contact residues of PfRON2 and PfAMA1. These structural and functional studies on the inhibitory peptides identified key interacting residues of PfAMA1 and PfRON2. PfRON2 peptide showed strain-independent strong erythrocyte invasion inhibitory activity that was not affected by contacts with some AMA1 polymorphic residues in contrast to R1 peptide that showed strong invasion inhibitory activity dependent on AMA1 polymorphism [32]. High throughput screening identified small molecule inhibitors of PfAMA1-PfRON2 interaction, which also inhibited RBC invasion* in vitro* [33]. This study attempts to use the structural determinants of crucial PfRON2 residues implicated in binding with PfAMA1 to identify peptidomimetics binding to PfAMA1 hydrophobic groove using* in silico* methods. Small molecules with structural similarity to peptidomimetics were identified that bound to the hydrophobic groove in blind docking.

## 2. Methodology

### 2.1. *De Novo *Structure Prediction of Peptides

*De novo* 3-dimensional structures of the peptides F1, F2, and F3 were predicted by PEP-FOLD server. By default, the program runs 100 simulations for an amino acid sequence and provides the best conformation of the five best clusters. Structure prediction and folding of the peptide are assumed for neutral pH [34].

### 2.2. Docking of Peptides on PfAMA1

The modeled peptides were docked on the hydrophobic groove containing region of PfAMA1 (PDB ID: 1Z40) with AutoDock Vina program (Molecular Graphics Laboratory) [35] on Windows platform. The best models of the peptides with zero root mean square deviation (RMSD) values were used for docking studies. To prepare the receptor for docking, PDB file of the receptor was opened in AutoDock Vina, water molecules were removed, polar hydrogens were added, and the receptor was saved as PDBQT file. To prepare the ligand, PDB file of the ligand was opened in AutoDock Vina and saved as PDBQT file. Grid parameters used for docking were 54 Å × 30 Å × 56 Å.

### 2.3. Virtual Screening of Peptidomimetic Inhibitors

Virtual screening of peptidomimetics against the crucial residues of PfAMA1 implicated in binding with PfRON2 peptide (residues Pro-2033, Phe-2038 to Arg-2041, and Pro-2044) was carried out with pepMMsMIMIC server [36]. This server is based on multiconformer, three-dimensional (3D) similarity search using as input the 3D structure of a peptide bound to a protein and suggests which chemical structures are able to mimic this natural interaction. Scoring method used was fingerprint-based filtering of shape similarity taking the side chain interactions into consideration.

### 2.4. Docking of Hits Obtained by Virtual Screening on PfAMA1

Five top-scoring peptidomimetic hits obtained by virtual screening were docked on PfAMA1 central region (PDB ID: 1Z40) hydrophobic groove containing region as the search base with AutoDock Vina program [35] on Windows operating system. Grid parameters used for docking were 54 Å × 30 Å × 56 Å. Similarly, the small drug-like compounds with structural similarity with the top 5 peptidomimetics were blindly docked on the whole molecule surface of PfAMA1 central region (PDB ID: 1Z40).

## 3. Results and Discussion

### 3.1. Phage Display Peptides (F1, F2, and F3) Dock on the Hydrophobic Groove of PfAMA1

**Figure 1:**
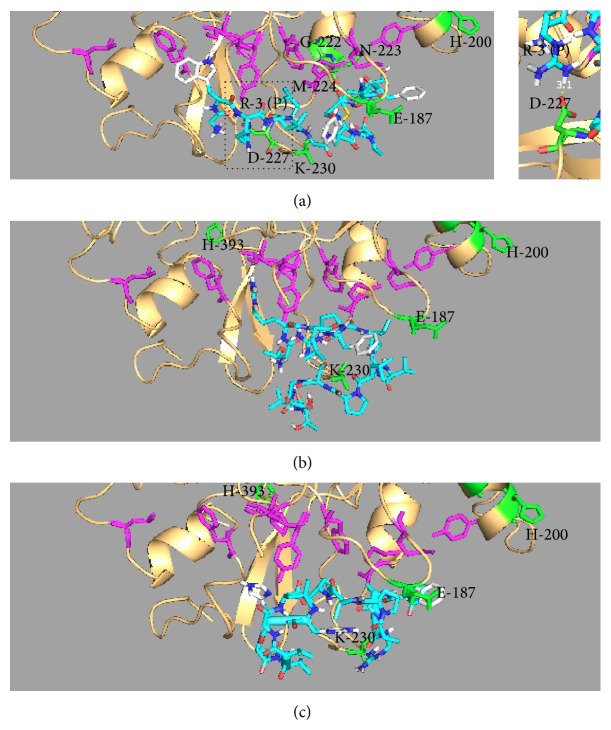
Docked structures of peptides F1 (a), F2 (b), and F3 (c) on PfAMA1 central region. PfAMA1 central region is shown in cartoon representation in orange with hydrophobic groove residues shown as sticks in magenta. Polymorphic residues are shown in stick representation in green. Ligands (peptides F1, F2, and F3) are shown in line representation. Peptide aliphatic carbon, oxygen, nitrogen, and aromatic carbon are shown in cyan, red, blue, and white, respectively. The ionic bond between R-3 of F1 peptide (R-3-P) and D-227 of PfAMA1 with bond length of 3.1 Å is shown in (a) inset. D-227 of PfAMA1 is shown in stick representation with carbon, oxygen, and nitrogen shown in green, red, and blue, respectively. Structures were visualized by molecular visualization software PyMOL (DeLano Scientific).

The hydrophobic groove on PfAMA1 is lined by residues Val-169, Phe-183, Met-190, Tyr-202, Met-224, Tyr-251, Ile-252, Leu-357, and Phe-367 [23] and constitutes the binding sites for R1 and RON2 peptides [32]. The solution state NMR structures of F1 and its variants and F2 peptides have been analyzed but there are no reports on their exact binding sites, although competition ELISA suggested that these peptides share the same binding site as R1 [25, 26]. To understand the molecular basis of binding of these peptides and their specificities, molecular docking approach was employed. Modeled peptides were docked on the region of PfAMA1 containing the hydrophobic groove. Peptide F1 bound to the expected hydrophobic groove site (Figure 1(a)). In PfRON2 peptide-PfAMA1 complex, Arg-2041 of the peptide is fitted in a pocket formed by residues Gly-222, Asn-223, Met-224, Ser-232, and Tyr-234 [32]. In case of PfAMA1 docked with F1 peptide, this pocket is absent, although the C-terminus of the peptide is in proximity with Gly-222, Asn-223, and Met-224 (Figure 1(a)). This interaction is also absent in PfAMA1-F2 complex (Figure 1(b)), whereas these three residues are in close proximity with N-terminus of F3 peptide in PfAMA1-F3 complex (Figure 1(c)). Arg-2041 of PfRON2 peptide is an important binding residue to PfAMA1 and fits in a well-formed pocket. Arg-15 of R1 fits in the same pocket as Arg-2041 of PfRON2 peptide [32]. This interaction was absent in PfAMA1-F1 complex, which may account for its lower affinity and invasion inhibitory activity. Lys-11 of R1 peptide makes an ionic bond with Asp-227 of PfAMA1. Although this ionic interaction is absent from PfRON2 peptide-PfAMA1 complex [32], a similar interaction between Arg-3 of F1 peptide and Asp-227 of PfAMA1 was observed (Figure 1(a) inset). Overall, although the sequences of F1, F2, and F3 peptides are unrelated to R1 and PfRON2 peptides, they also bind to the hydrophobic region of PfAMA1 and are likely to act via similar mechanism.

### 3.2. Identification of Peptidomimetics Targeting the Hydrophobic Groove of PfAMA1

**Figure 2:**
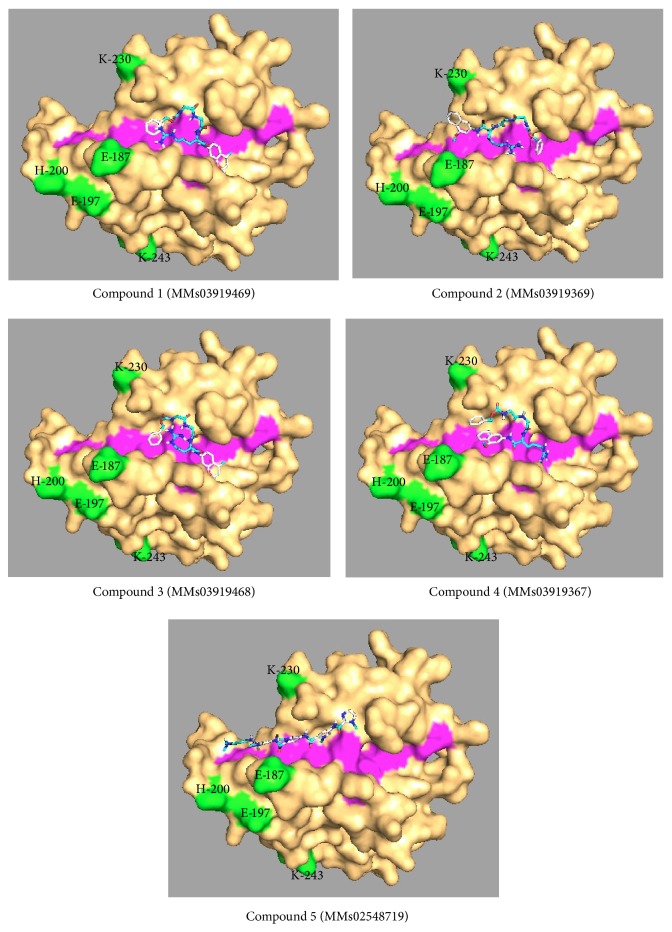
Docked structures of top 5 peptidomimetic hits binding to the hydrophobic groove of PfAMA1 central region. PfAMA1 central region is shown in surface representation in orange, with hydrophobic groove shown in magenta and polymorphic residues shown in green. Structures were visualized by molecular visualization software PyMOL (DeLano Scientific).

In the cocrystal structures with PfAMA1, PfRON2sp1 and R1-major peptides showed significant structural similarity (PfRON2sp1, Ala-2031 to Met-2042; R1-major, Phe-P5 to Met-P16). R1-major also showed mimicry with PfRON2 peptide in the cystine loop-binding region (Phe-2038/Phe-P12 to Arg-2041/Arg-P15) [32]. The residues Pro-2033, Phe-2038 to Arg-2041, and Pro-2044 from PfRON2 played prominent role in binding with PfAMA1, so they were selected for identification of peptidomimetics targeting the PfAMA1-PfRON2 binding interface of PfAMA1 with pepMMsMIMIC server. Table S1 in Supplementary Material available online at http://dx.doi.org/10.1155/2014/642391 presents the list of top 50 peptidomimetics obtained by virtual screening. Top 5 hits were docked on PfAMA1 region containing the hydrophobic groove and found to bind at the hydrophobic groove (Figure 2).

### 3.3. Identification of Drug-Like Molecules Binding to the Hydrophobic Groove

**Figure 3:**
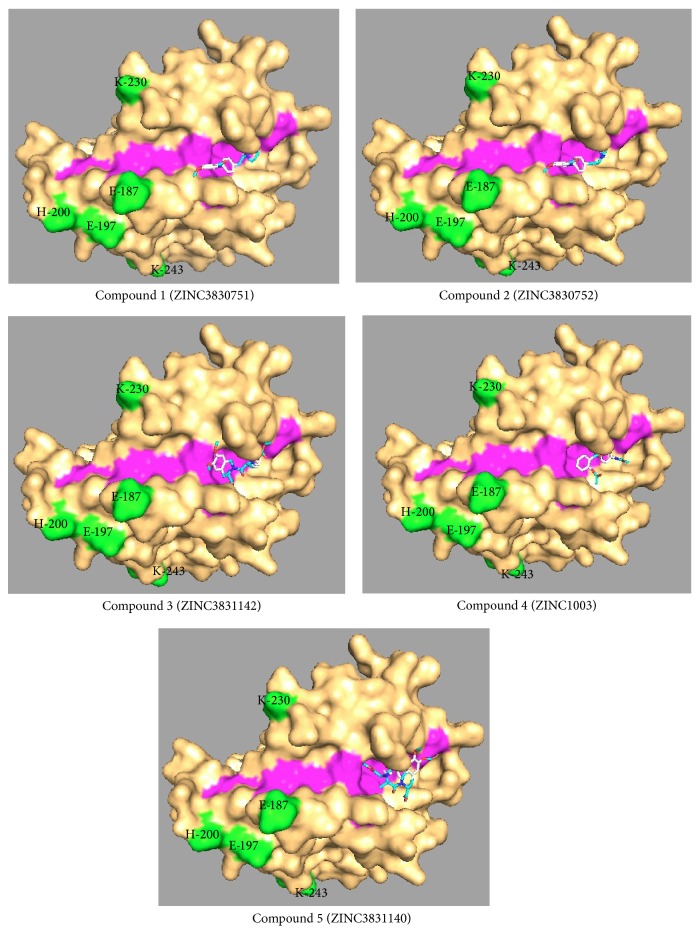
Docked structures of top 5 small drug-like molecules binding to the hydrophobic groove upon blind docking on the whole molecule surface of PfAMA1 central region. PfAMA1 central region is shown in surface representation in orange, with hydrophobic groove shown in magenta and polymorphic residues shown in green. Structures were visualized by molecular visualization software PyMOL (DeLano Scientific).

Small drug-like molecules having structural similarity to peptidomimetic hits 1 to 5 were selected on the basis of Lipinski's rule of five to predict drug-likeness [37]. Lipinski's criteria are based on data in the literature for a large number of compounds, including all known drugs correlating physical properties with their bioavailability. The molecules violating Lipinski's rule of five were excluded. Table S2 presents small drug-like molecules having structural similarity with peptidomimetics 1 to 5. To check the specificity of these molecules for binding to the hydrophobic groove of PfAMA1, these molecules were blindly docked on the whole molecule surface of PfAMA1 central region (PDB ID: 1Z40). The molecules, which bound to the hydrophobic groove, are presented in Table 1 in the order of their binding affinities. Complexes of top five of the docked molecules with PfAMA1 central region are shown in Figure 3 and the zoomed views of the bound ligands are shown in Figure S1. These molecules were not found to have any contact with polymorphic residues suggesting their strain-independent action.

Functions of proteins depend on their interacting partners to a great extent both under normal and pathological conditions. Protein complexes involved in pathogen-specific processes constitute potential drug targets. Although it is not always possible to target the protein interfaces because of the involvement of large surface area, it has been recognized that in many cases protein interfaces could be targeted because of the presence of hot spots composed of crucial binding residues (reviewed by Buchwald 2010) [38].

The interaction between PfAMA1 and PfRON2 has been thoroughly studied and the blockade of this interaction by drug-like molecules has been shown to result in invasion inhibition, underscoring the potential of this interaction as a promising antimalarial drug target [33]. This study uses* in silico* tools to identify the potential blockers of this interaction based on the structural determinants of crucial PfAMA1-binding residues. The peptidomimetic hits were docked on a defined area of PfAMA1 encompassing the hydrophobic groove to avoid nonspecific binding at other regions of the protein due to their large size and flexibility. However, the drug-like compounds based on peptidomimetics were docked blindly on the whole molecule surface and bound specifically to the hydrophobic groove suggesting their potential to inhibit the interaction.

## 4. Conclusion

To control malaria, an effective vaccine and novel drugs are urgently needed. Molecules involved in merozoite invasion of host RBCs have been a major focus of drug target discovery. PfAMA1, being crucial for RBC invasion, is an attractive candidate to be targeted for invasion inhibition. A peptide derived from PfRON2, a component of RON complex that directly interacts with PfAMA1, inhibited RBC invasion* in vitro*, hence presenting an attractive design for development of invasion inhibitory molecules targeting PfAMA1. This study bioinformatically identified some small drug-like molecules based on the structures of peptidomimetic molecules selected with crucial PfAMA1-binding residues of PfRON2 as template. The selected molecules were tested for their binding specificity of the whole molecule surface of PfAMA1 central region and found to bind to the expected hydrophobic groove. These drug-like molecules could be tested for* in vitro* binding to PfAMA1 and could be potential invasion inhibitory lead molecules for development of future antimalarials.

## Supplementary Material

Table S1: List of top 50 peptidomimetic compounds obtained by virtual screening against 6-residues (Pro-2033, Phe-2038 to Arg-2041 and Pro-2044) from PfRON2 peptide with pepMMsMIMIC server.
Table S2: List of compounds targeting hydrophobic groove of PfAMA1 and having structural similarity with top 5 peptidomimetics obtained by virtual screening with pepMMsMIMIC server .
Fig. S1. Zoomed view of docked structures of top 5 small drug-like molecules on the hydrophobic groove of PfAMA1.

## Figures and Tables

**Table 1:** List of small drug-like molecules binding to the hydrophobic groove of PfAMA1 based on the structures of PfAMA1-binding peptidomimetics.

S. number	Compound (ZINC ID)	Binding affinity (kcal/mol)	Properties
1	ZINC03830751	−7.6	Mol. Wt.: 352.478 *x*log⁡*P*: 4.36 Charge: 0 H-bond donor: 1 H-bond acceptor: 3

2	ZINC03830752	−7.1	Mol. Wt.: 352.478 *x*log⁡*P*: 4.36 Charge: 0 H-bond donor: 1 H-bond acceptor: 3

3	ZINC03831142	−7.0	Mol. Wt.: 498.576 *x*log⁡*P*: 2.85 Charge: 0 H-bond donor: 3 H-bond acceptor: 7

4	ZINC00001003	−6.9	Mol. Wt.: 313.309 *x*log⁡*P*: 2.79 Charge: 0 H-bond donor: 1 H-bond acceptor: 3

5	ZINC03775140	−6.9	Mol. Wt.: 465.953 *x*log⁡*P*: 3.36 Charge: 0 H-bond donor: 2 H-bond acceptor: 5

6	ZINC03831143	−6.8	Mol. Wt.: 498.576 *x*log⁡*P*: 2.85 Charge: 0 H-bond donor: 3 H-bond acceptor: 7

7	ZINC03831141	−6.8	Mol. Wt.: 498.576 *x*log⁡*P*: 2.85 Charge: 0 H-bond donor: 3 H-bond acceptor: 7

8	ZINC00608261	−6.7	Mol. Wt.: 373.884 *x*log⁡*P*: 3.59 Charge: 0 H-bond donor: 2 H-bond acceptor: 3

9	ZINC03813069	−6.4	Mol. Wt.: 379.501 *x*log⁡*P*: 2.89 Charge: H-bond donor: 3 H-bond acceptor: 5

10	ZINC00000128	−6.2	Mol. Wt.: 292.379 *x*log⁡*P*: 4.36 Charge: 0 H-bond donor: 3 H-bond acceptor: 4

11	ZINC02001884	−5.6	Mol. Wt.: 379.501 *x*log⁡*P*: 2.89 Charge: 0 H-bond donor: 3 H-bond acceptor: 5

## Abbreviations

AMA1: Apical membrane antigen 1
RON2: Rhoptry neck protein 2
NMR: Nuclear magnetic resonance.
