# Supplementary material for: Bioinformatic Identification of Peptidomimetic-Based Inhibitors against Plasmodium falciparum Antigen AMA1
Source: Malar Res Treat. 2014 Dec 18;2014:642391. doi: 10.1155/2014/642391 (PMC4281401; doi:10.1155/2014/642391)
Supplement: Supplementary file 1 — Table S1: List of top 50 peptidomimetic compounds obtained by virtual screening against 6-residues (Pro-2033, Phe-2038 to Arg-2041 and Pro-2044) from PfRON2 peptide with pepMMsMIMIC server. Table S2: List of compounds targeting hydrophobic groove of PfAMA1 and having structural similarity with top 5 peptidomimetics obtained by virtual screening with pepMMsMIMIC server . Fig. S1. Zoomed view of docked structures of top 5 small drug-like molecules on the hydrophobic groove of PfAMA1. [file 642391.f1.zip › 642391.f1/Table S1.pdf]

Table S1

S. No.	pepMMsMIMIC Compound	Score	Mol. Wt.	Structure
1	MMs03919469	0.55	580.622	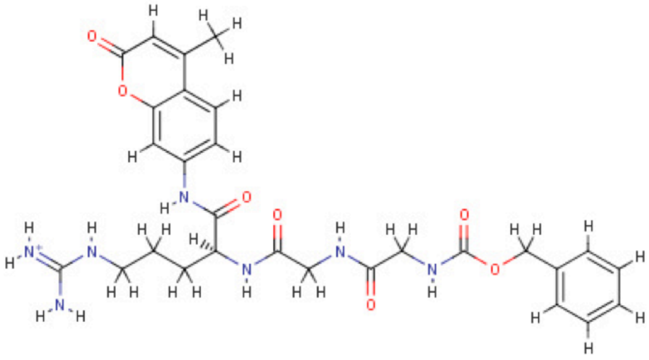
2	MMs03919369	0.54	578.65	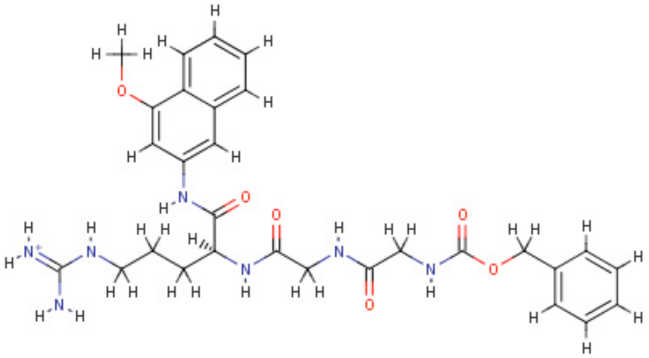
3	MMs03919468	0.50	579.614	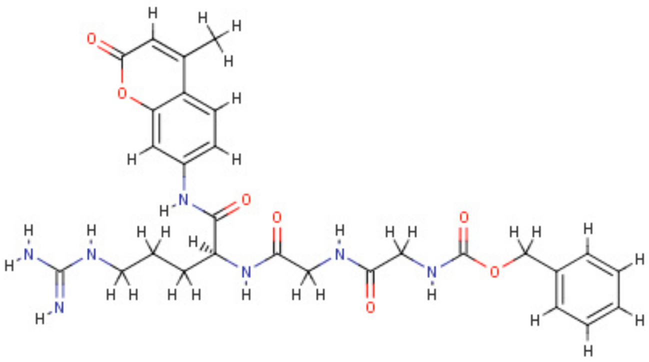

4	MMs03919367	0.49	578.65	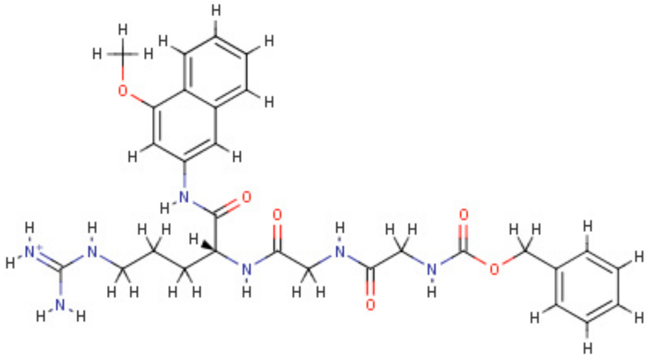
5	MMs02548719	0.49	577.67	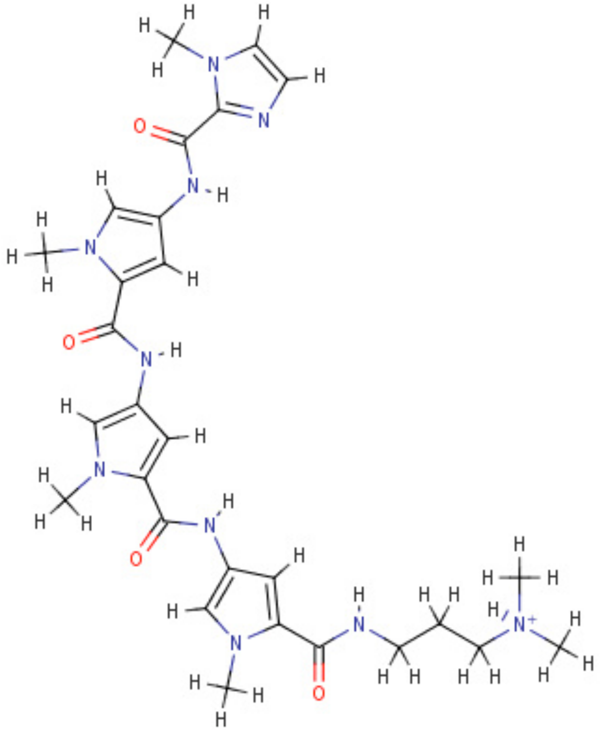

6	MMs03918861	0.49	664.692	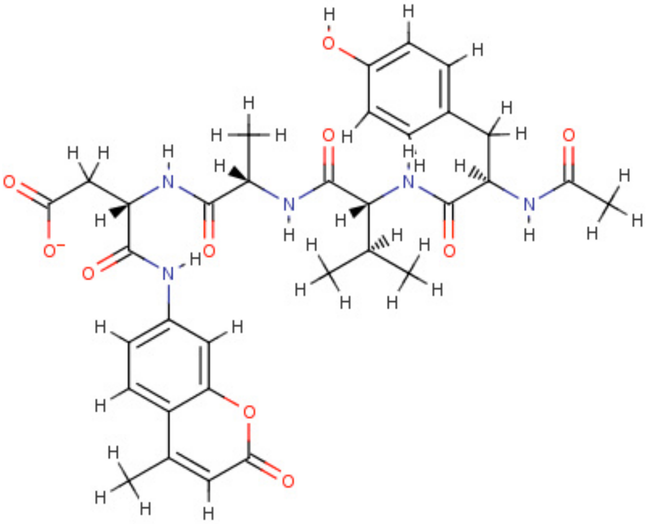
7	MMs03919366	0.45	577.642	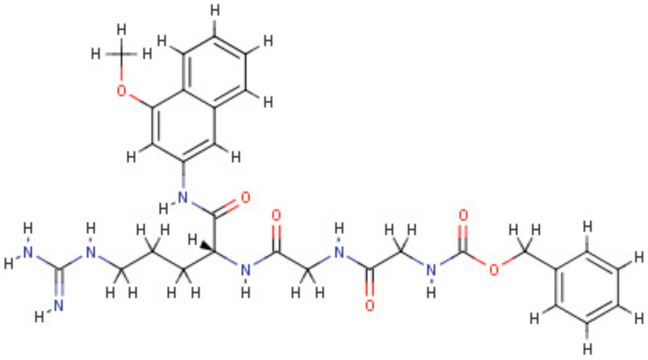
8	MMs03919425	0.45	548.624	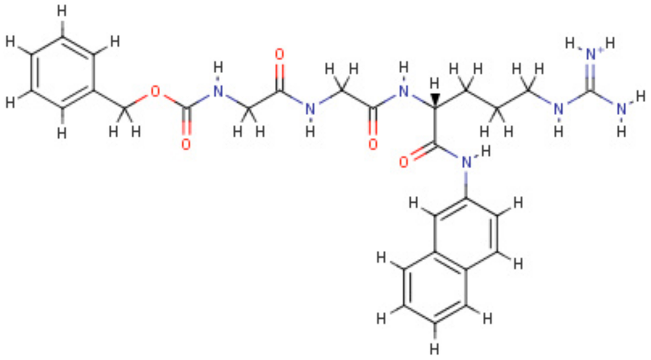

9	MMs03919422	0.45	547.616	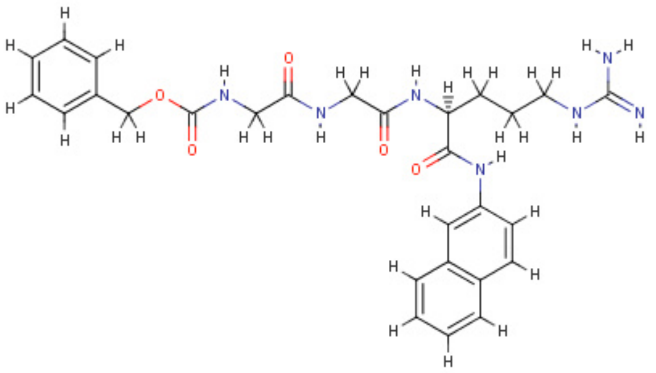
10	MMs03919424	0.44	547.616	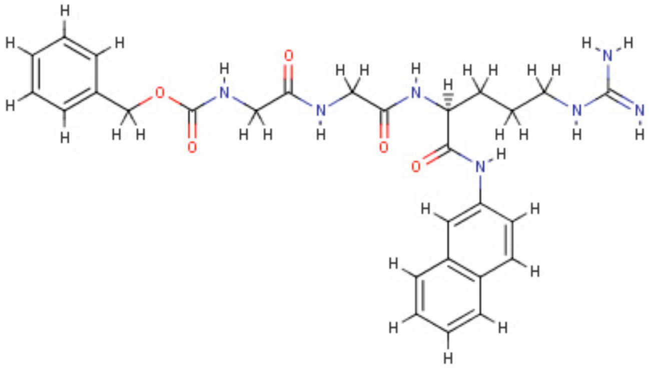
11	MMs03924092	0.43	730.9	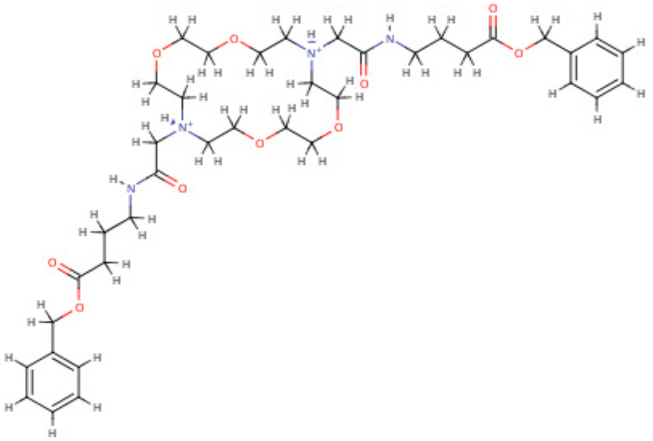

12	MMs03919433	0.42	588.618	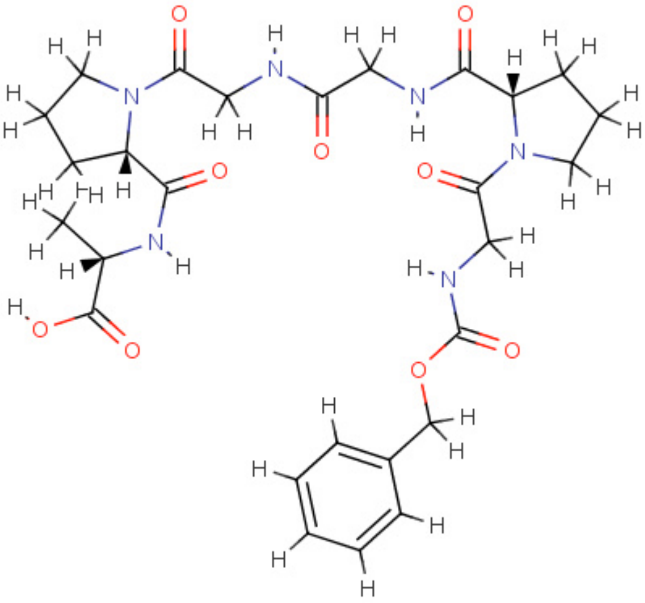
13	MMs03919423	0.41	548.624	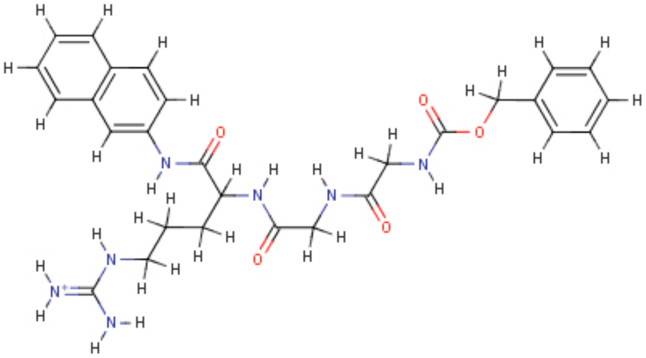
14	MMs03924085	0.40	674.792	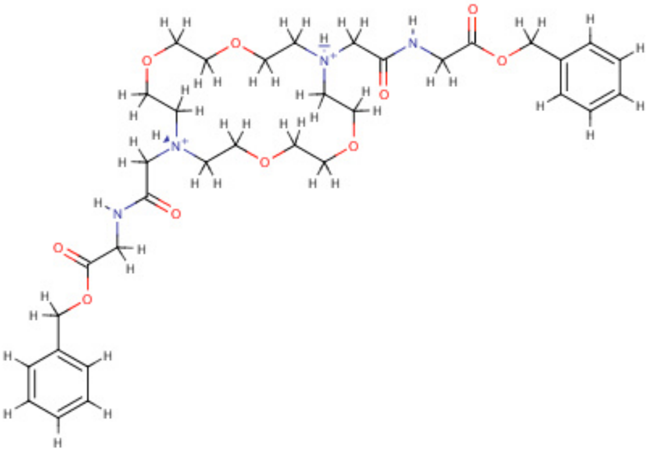

15	MMs03919467	0.39	580.622	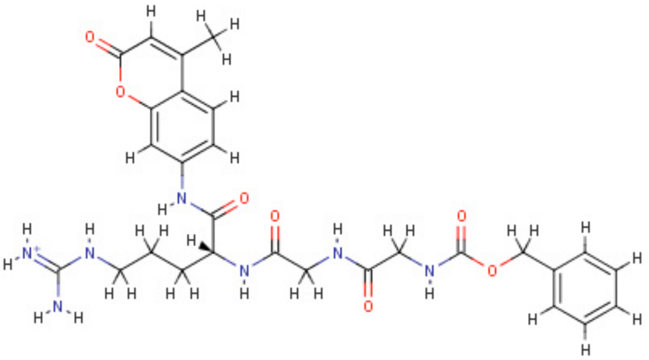
16	MMs03918885	0.37	628.639	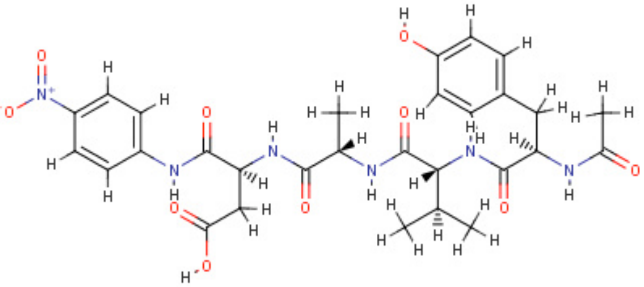
17	MMs02467784	0.36	587.722	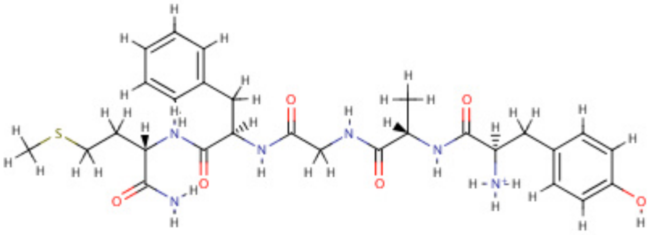

18	MMs03918860	0.36	665.7	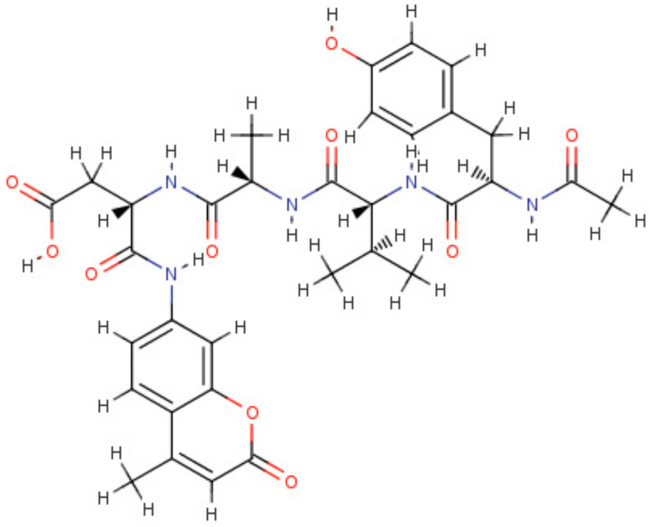
19	MMs03919368	0.36	577.642	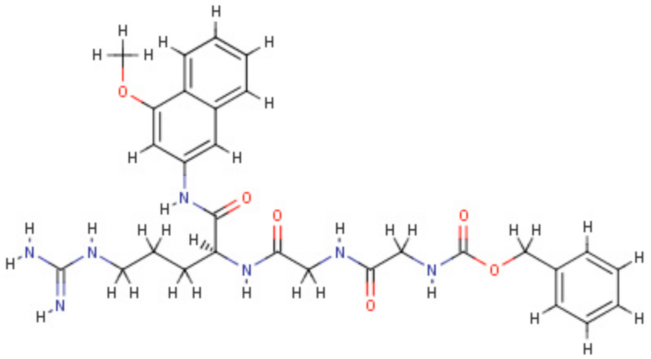

20	MMs03919466	0.36	579.614	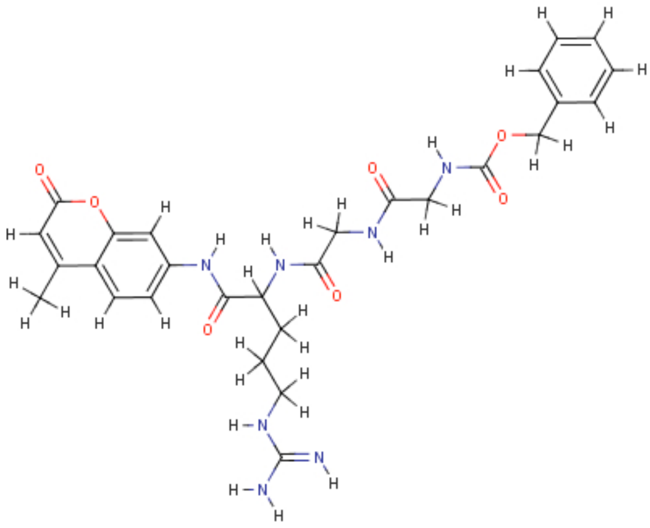
21	MMs03918883	0.32	628.639	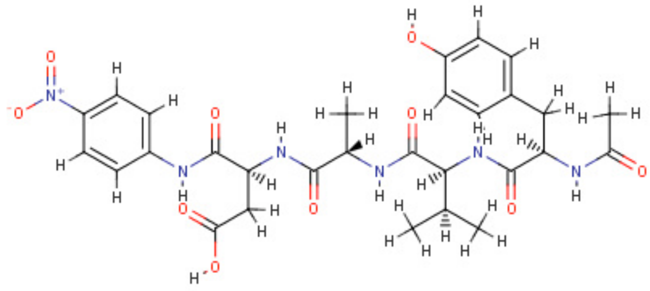
22	MMs03918889	0.30	628.639	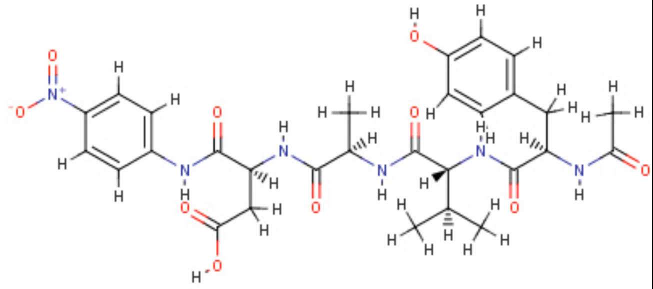

23	MMs01727547	0.29	812.886	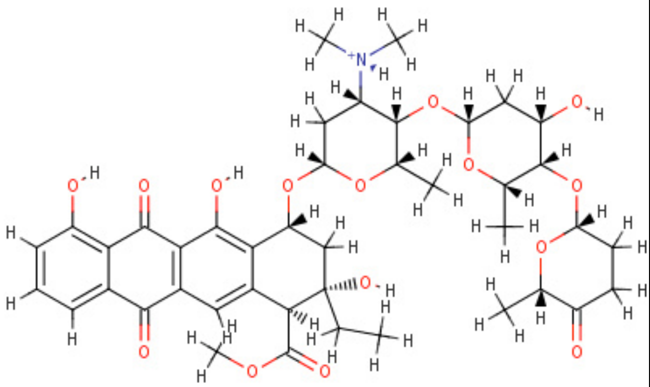
24	MMs02467779	0.29	586.714	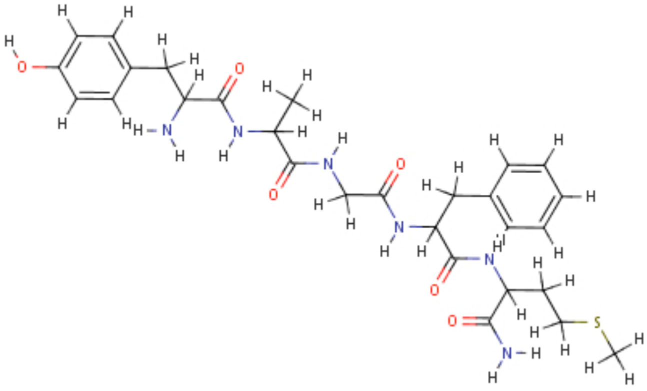
25	MMs02467780	0.28	587.722	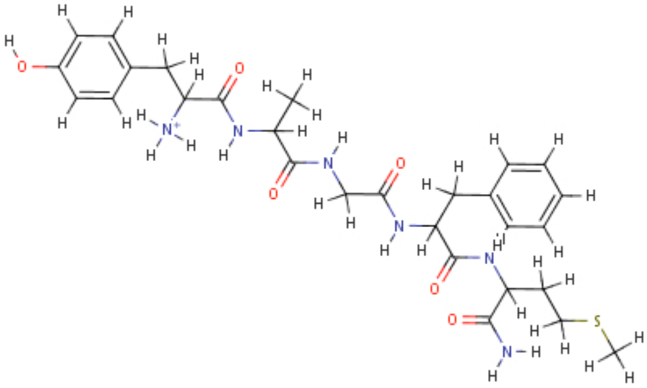

26	MMs02391032	0.27	604.749	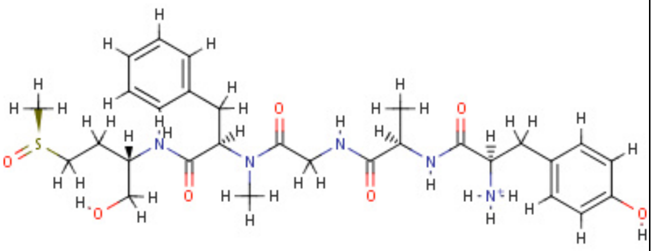
27	MMs03918857	0.27	664.692	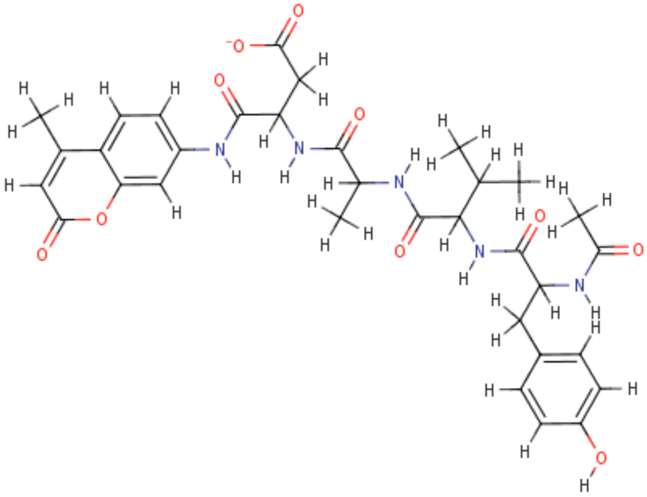

28	MMs03079507	0.27	486.517	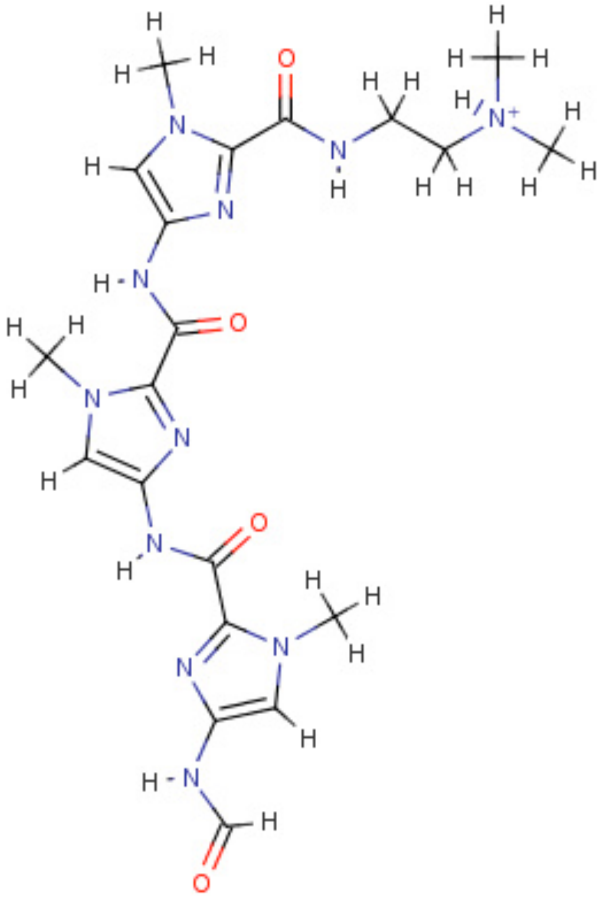
29	MMs01094012	0.26	565.624	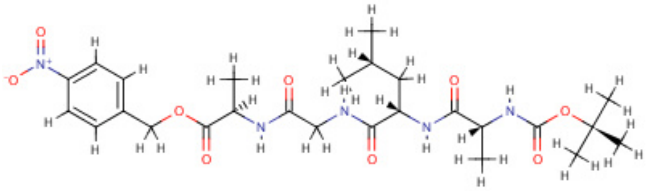
30	MMs03918890	0.26	627.631	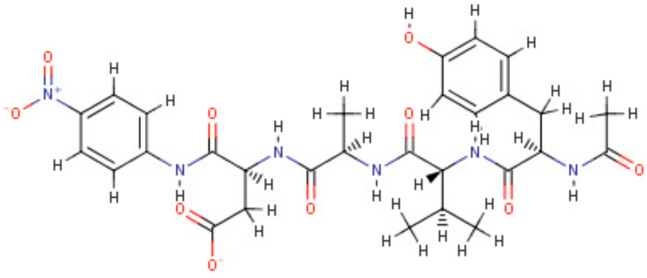

31	MMs01727549	0.25	812.886	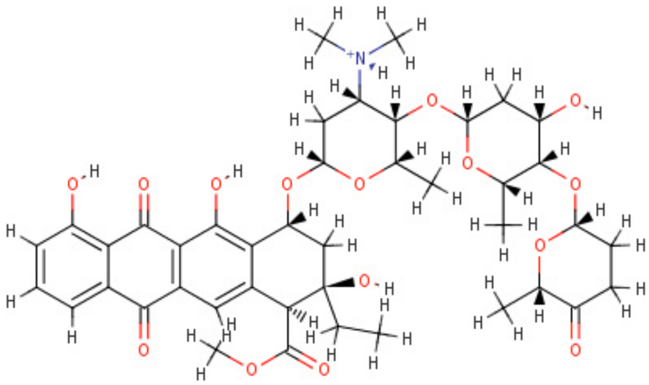
32	MMs03919145	0.25	601.705	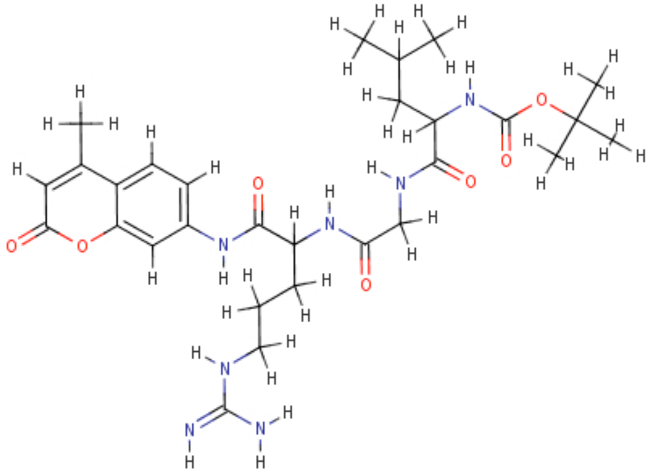
33	MMs02467786	0.25	587.722	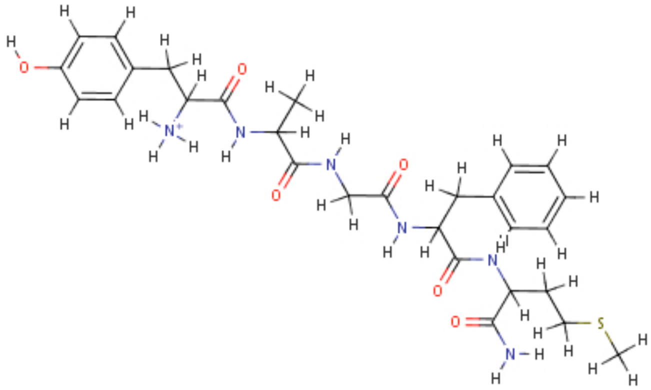

34	MMs02391028	0.25	604.749	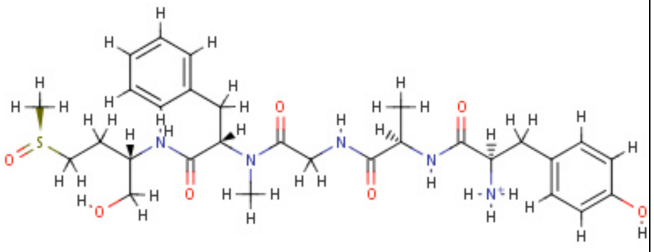
35	MMs02489379	0.25	611.477	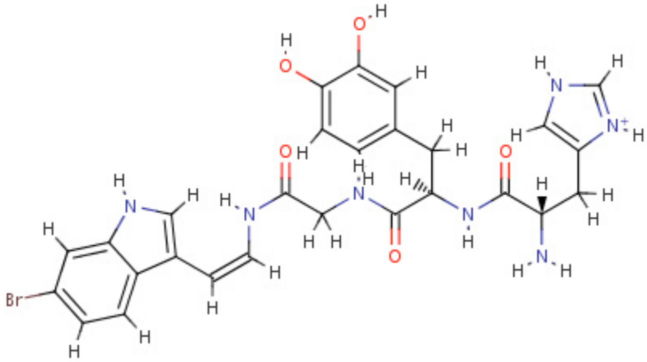
36	MMs02481161	0.24	542.617	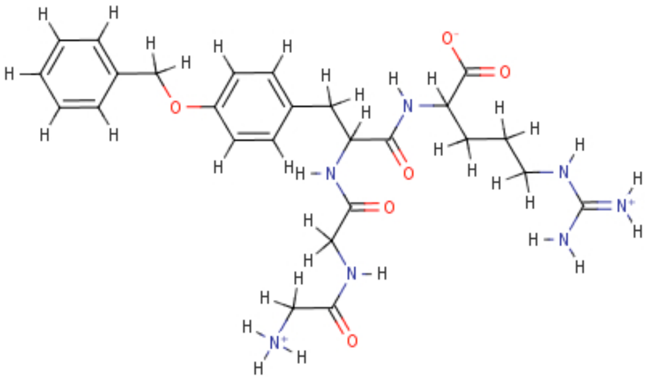
37	MMs02514534	0.24	569.683	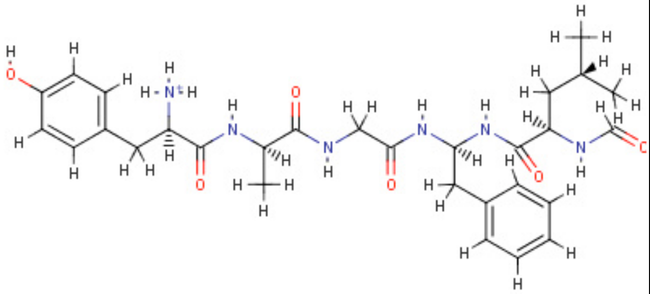

38	MMs02467782	0.24	587.722	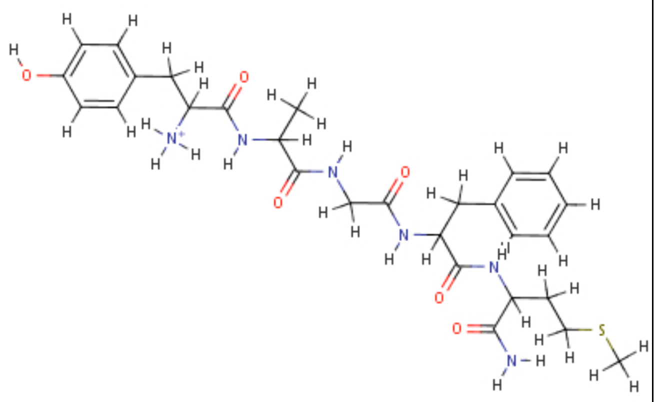
39	MMs03919122	0.23	573.675	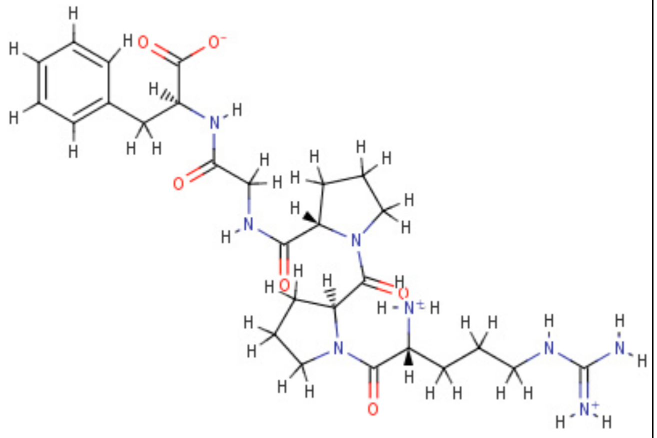
40	MMs02514549	0.23	569.683	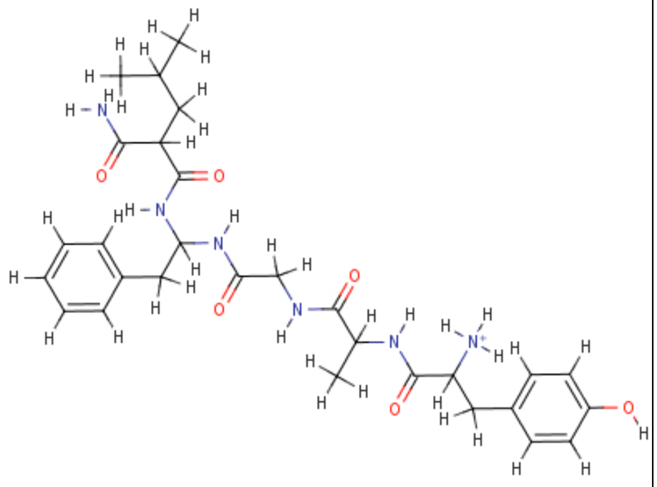

41	MMs02391026	0.23	604.749	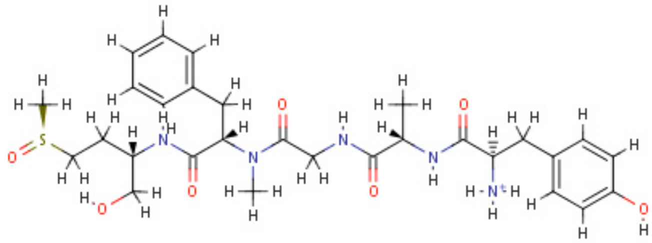
42	MMs02514564	0.23	569.683	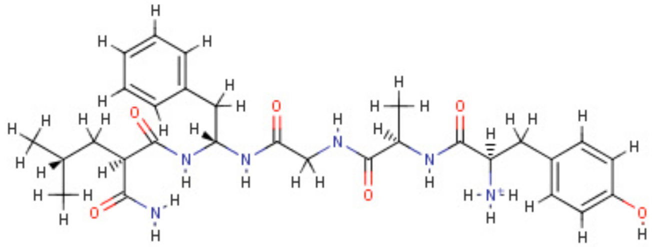
43	MMs03919120	0.23	616.78	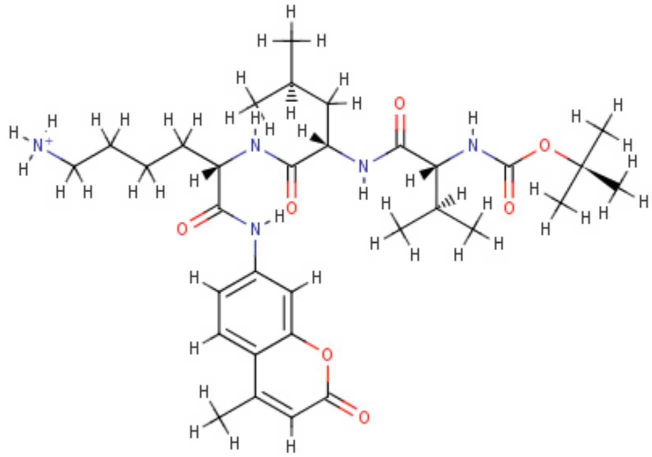

44	MMs03919124	0.22	573.675	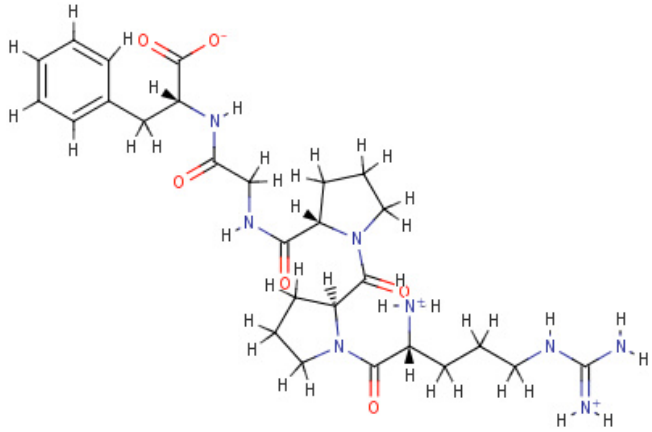
45	MMs03919146	0.22	602.713	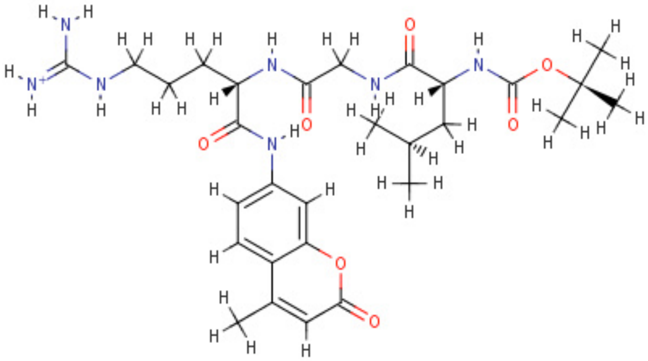
46	MMs02514537	0.22	569.683	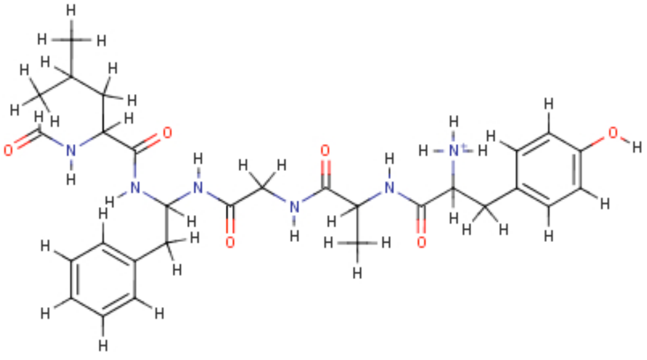

47	MMs03919198	0.22	681.794	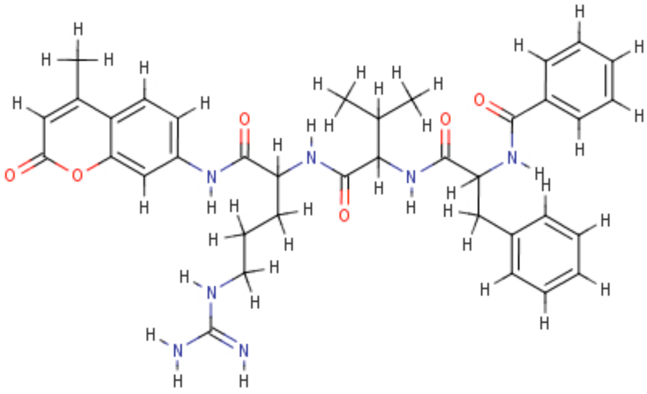
48	MMs02514563	0.22	569.683	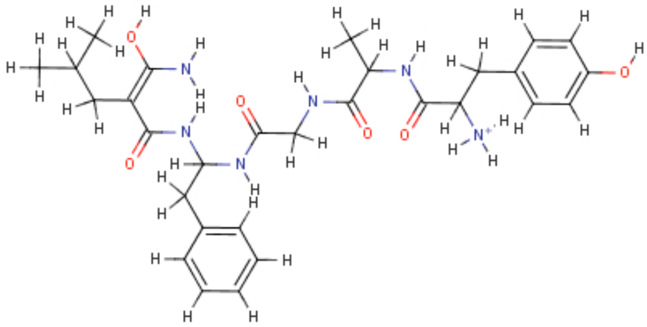
49	MMs02421858	0.21	623.711	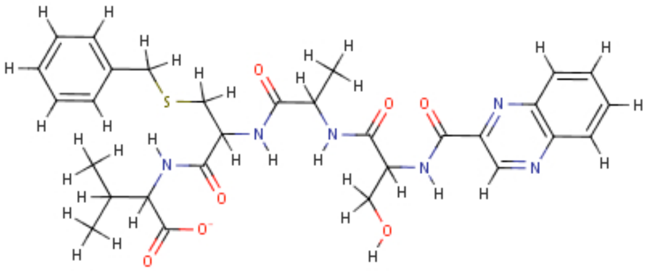
50	MMs02487107	0.21	526.643	

				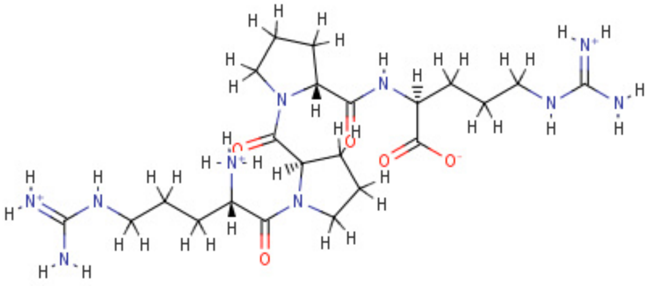
