# Supplementary material for: Bioinformatic Identification of Peptidomimetic-Based Inhibitors against Plasmodium falciparum Antigen AMA1
Source: Malar Res Treat. 2014 Dec 18;2014:642391. doi: 10.1155/2014/642391 (PMC4281401; doi:10.1155/2014/642391)
Supplement: Supplementary file 1 — Table S1: List of top 50 peptidomimetic compounds obtained by virtual screening against 6-residues (Pro-2033, Phe-2038 to Arg-2041 and Pro-2044) from PfRON2 peptide with pepMMsMIMIC server. Table S2: List of compounds targeting hydrophobic groove of PfAMA1 and having structural similarity with top 5 peptidomimetics obtained by virtual screening with pepMMsMIMIC server . Fig. S1. Zoomed view of docked structures of top 5 small drug-like molecules on the hydrophobic groove of PfAMA1. [file 642391.f1.zip › 642391.f1/Table S2.pdf]

Table S2

pepMMsMIMIc hit	Similar drug-like molecule (ZINC ID)	Compound 2D structure	Properties
MMs03919469	ZINC00896463	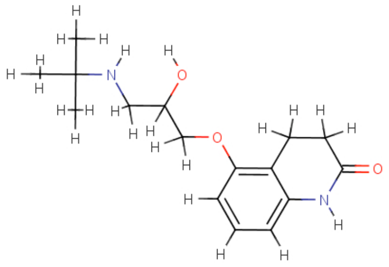	Mol. Wt.: 292.379<br>xLogP: 1.70<br>Charge: 0<br>H-bond donor: 3<br>H-bond acceptor: 4
MMs03919469	ZINC00000128	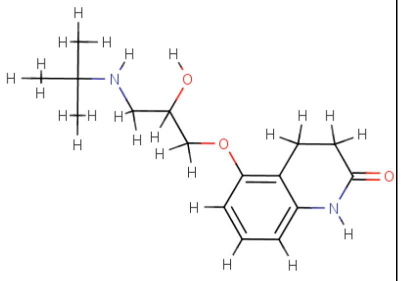	Mol. Wt.: 292.379<br>xLogP: 4.36<br>Charge: 0<br>H-bond donor: 3<br>H-bond acceptor: 4
MMs03919469	ZINC03830752	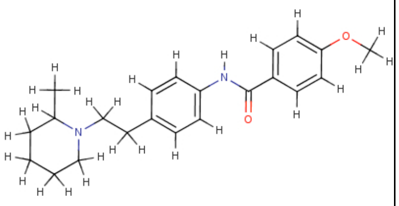	Mol. Wt.: 352.478<br>xLogP: 4.36<br>Charge: 0<br>H-bond donor: 1<br>H-bond acceptor: 3
MMs03919469	ZINC03830751	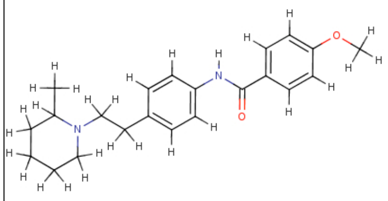	Mol. Wt.: 352.478<br>xLogP: 4.36<br>Charge: 0<br>H-bond donor: 1<br>H-bond acceptor: 3

MMs03919469	ZINC00001003	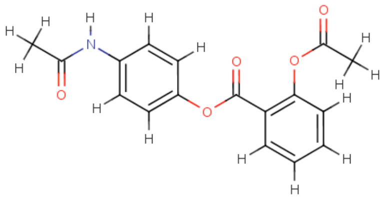	Mol. Wt.: 313.309<br>xLogP: 2.79<br>Charge: 0<br>H-bond donor: 1<br>H-bond acceptor: 3
MMs03919469	ZINC03813069	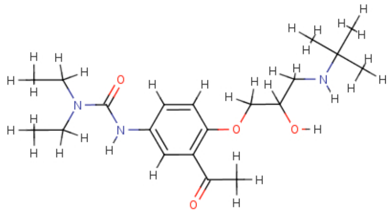	Mol. Wt.: 379.501<br>xLogP: 2.89<br>Charge:<br>H-bond donor: 3<br>H-bond acceptor: 5
MMs03919469	ZINC02001884	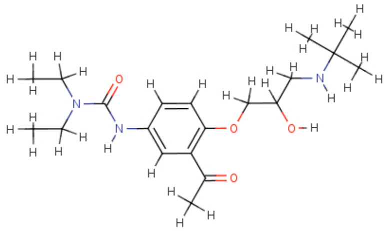	Mol. Wt.: 379.501<br>xLogP: 2.89<br>Charge: 0<br>H-bond donor: 3<br>H-bond acceptor: 5
MMs03919469	ZINC00608261	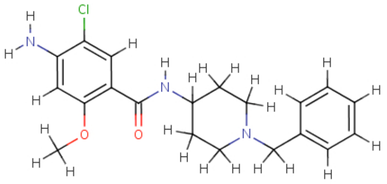	Mol. Wt.: 373.884<br>xLogP: 3.59<br>Charge: 0<br>H-bond donor: 2<br>H-bond acceptor: 3
MMs03919469	ZINC03830564	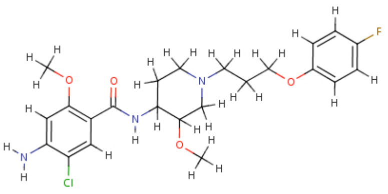	Mol. Wt.: 465.953<br>xLogP: 3.36<br>Charge: 0<br>H-bond donor: 2<br>H-bond acceptor: 5
MMs03919469	ZINC03775140	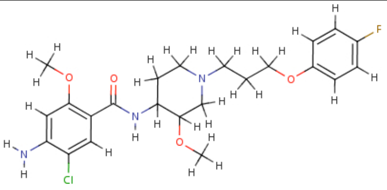	Mol. Wt.: 465.953<br>xLogP: 3.36<br>Charge: 0<br>H-bond donor: 2<br>H-bond acceptor: 5

MMs03919369	ZINC03830751	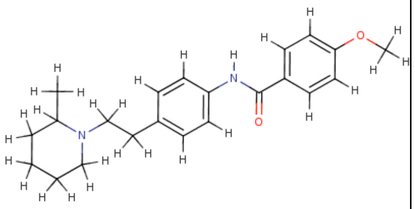	Mol. Wt.: 352.478<br>xLogP: 4.36<br>Charge: 0<br>H-bond donor: 1<br>H-bond acceptor: 3
MMs03919369	ZINC03830752	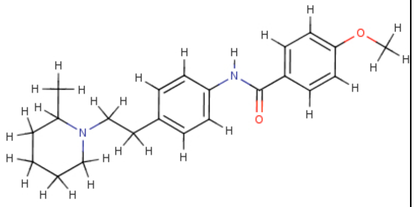	Mol. Wt.: 352.478<br>xLogP: 1<br>Charge: 0<br>H-bond donor: 1<br>H-bond acceptor: 3
MMs03919369	ZINC03831141	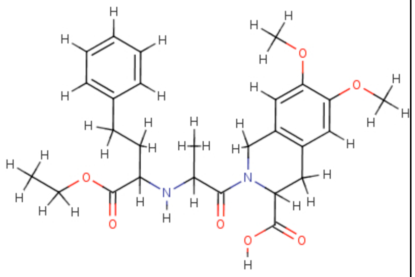	Mol. Wt.: 498.576<br>xLogP: 2.85<br>Charge: 0<br>H-bond donor: 3<br>H-bond acceptor: 7
MMs03919369	ZINC03831142	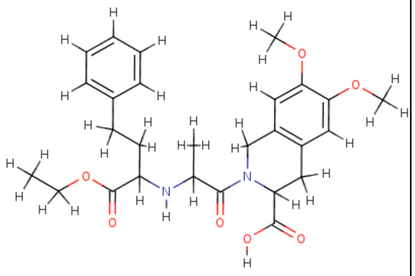	Mol. Wt.: 498.576<br>xLogP: 2.85<br>Charge: 0<br>H-bond donor: 3<br>H-bond acceptor: 7
MMs03919369	ZINC03831143	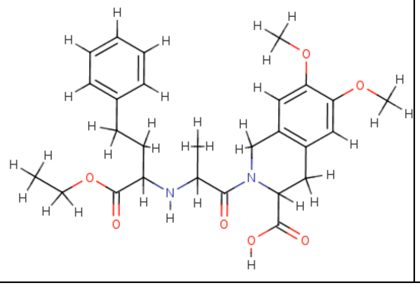	Mol. Wt.: 498.576<br>xLogP: 2.85<br>Charge: 0<br>H-bond donor: 3<br>H-bond acceptor: 7

MMs03919369	ZINC03831140	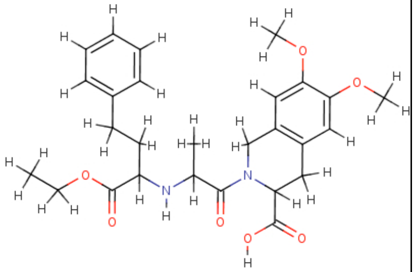	Mol. Wt.: 498.576<br>xLogP: 2.85<br>Charge: 0<br>H-bond donor: 3<br>H-bond acceptor: 7
MMs03919369	ZINC00000128	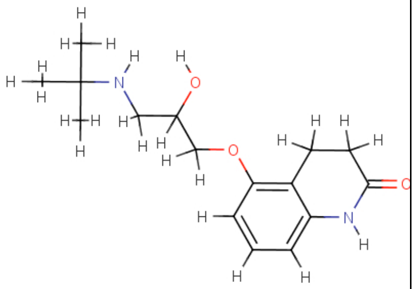	Mol. Wt.: 292.379<br>xLogP: 1.70<br>Charge: 0<br>H-bond donor: 3<br>H-bond acceptor: 4
MMs03919369	ZINC00896463	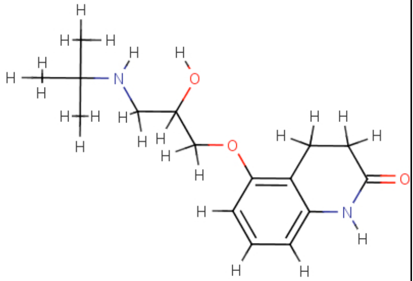	Mol. Wt.: 292.379<br>xLogP: 1.70<br>Charge: 0<br>H-bond donor: 3<br>H-bond acceptor: 4
MMs03919468	ZINC00000128	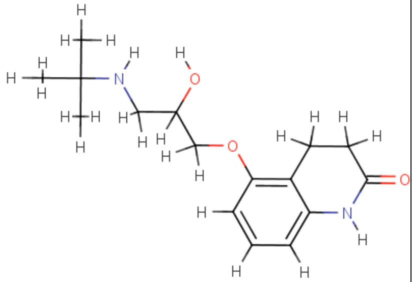	Mol. Wt.: 292.379<br>xLogP: 1.70<br>Charge: 0<br>H-bond donor: 3<br>H-bond acceptor: 4
MMs03919468	ZINC00001003	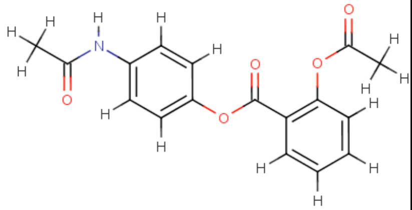	Mol. Wt.: 313.309<br>xLogP: 2.79<br>Charge: 0<br>H-bond donor: 1<br>H-bond acceptor: 3

MMs03919468	ZINC02001884	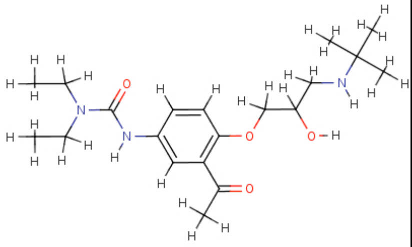	Mol. Wt.: 379.501<br>xLogP: 2.89<br>Charge: 0<br>H-bond donor: 3<br>H-bond acceptor: 5
MMs03919468	ZINC03775140	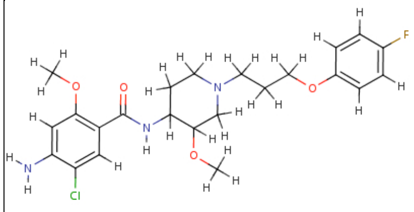	Mol. Wt.: 465.953<br>xLogP: 3.36<br>Charge: 0<br>H-bond donor: 2<br>H-bond acceptor: 5
MMs03919468	ZINC03830564	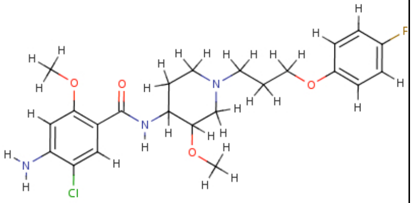	Mol. Wt.: 465.953<br>xLogP: 3.36<br>Charge: 0<br>H-bond donor: 2<br>H-bond acceptor: 5
MMs03919367	ZINC03830751	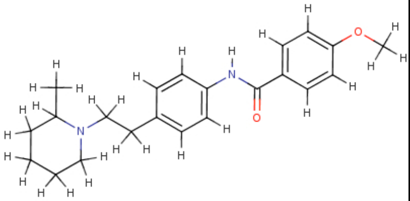	Mol. Wt.: 352.478<br>xLogP: 4.36<br>Charge: 0<br>H-bond donor: 1<br>H-bond acceptor: 3
MMs03919367	ZINC03830752	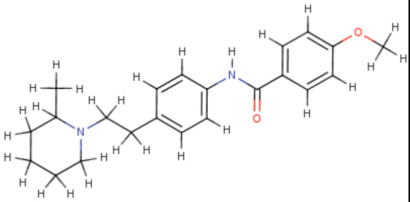	Mol. Wt.: 352.478<br>xLogP: 4.36<br>Charge: 0<br>H-bond donor: 1<br>H-bond acceptor: 3

MMs03919367	ZINC03831141	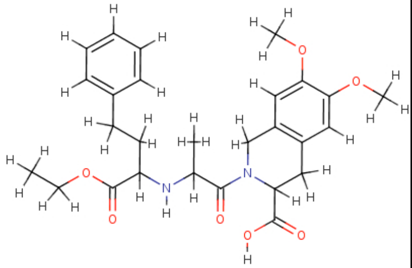	Mol. Wt.: 498.576<br>xLogP: 2.85<br>Charge: 0<br>H-bond donor: 3<br>H-bond acceptor: 7
MMs03919367	ZINC03831142	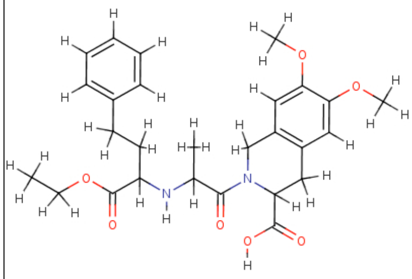	Mol. Wt.:<br>498.576<br>xLogP: 2.85<br>Charge:0<br>H-bond donor: 3<br>H-bond acceptor: 7
MMs03919367	ZINC03831143	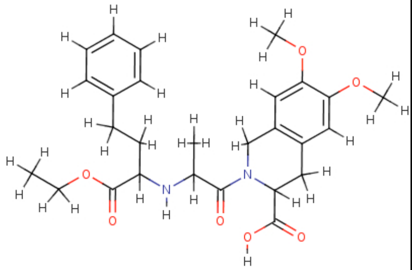	Mol. Wt.: 498.576<br>xLogP: 2.85<br>Charge: 0<br>H-bond donor: 3<br>H-bond acceptor: 7
MMs03919367	ZINC03831140	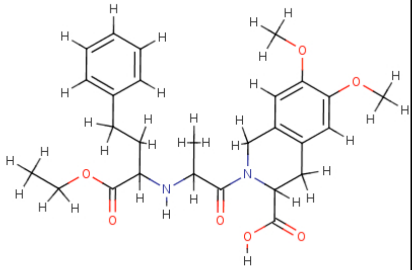	Mol. Wt.: 498.576<br>xLogP: 2.85<br>Charge: 0<br>H-bond donor: 3<br>H-bond acceptor: 7

MMs03919367	ZINC00000128	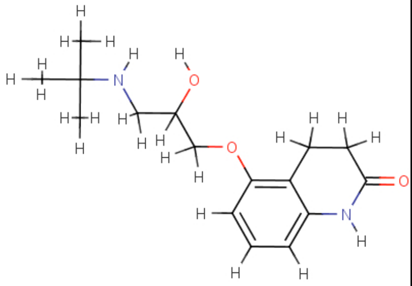	Mol. Wt.: 292.379<br>xLogP: 1.70<br>Charge: 0<br>H-bond donor: 3<br>H-bond acceptor: 4
MMs03919367	ZINC00896463	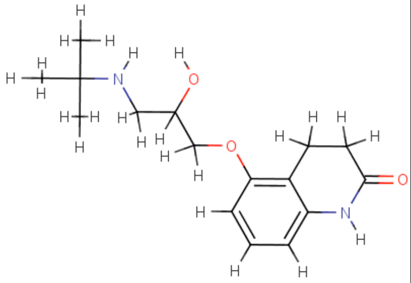	Mol. Wt.: 292.379<br>xLogP: 1.70<br>Charge: 0<br>H-bond donor: 3<br>H-bond acceptor: 4
MMs02548719	ZINC00606503	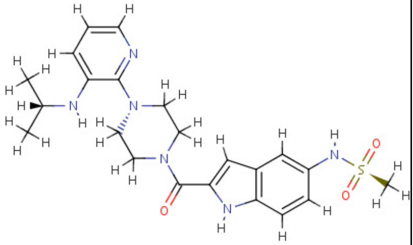	Mol. Wt.: 456.571<br>xLogP: 2.72<br>Charge: 0<br>H-bond donor: 3<br>H-bond acceptor: 4
