# Supplementary figures and images for: Bioinformatic Identification of Peptidomimetic-Based Inhibitors against Plasmodium falciparum Antigen AMA1
Source: Malar Res Treat. 2014 Dec 18;2014:642391. doi: 10.1155/2014/642391 (PMC4281401; doi:10.1155/2014/642391)

**Fig. S1**

**Compound 1**  
**(ZINC3830751)**

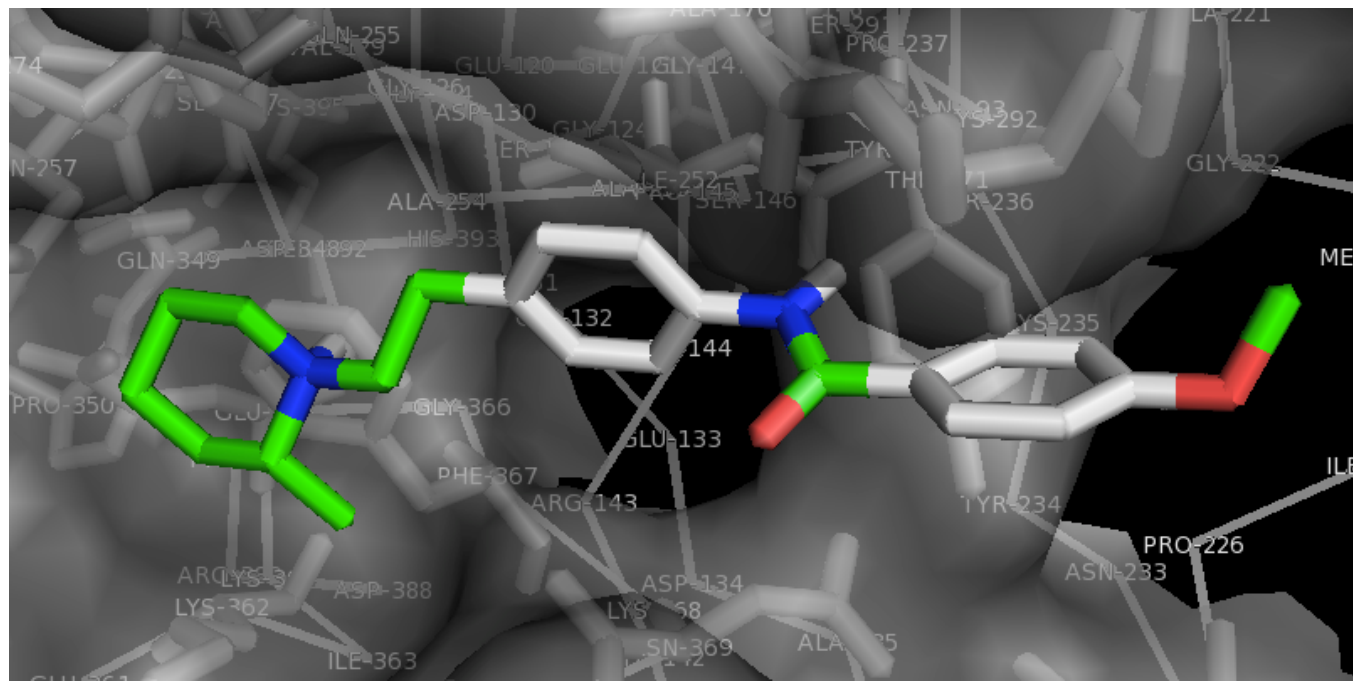

**Compound 2**  
**(ZINC3830752)**

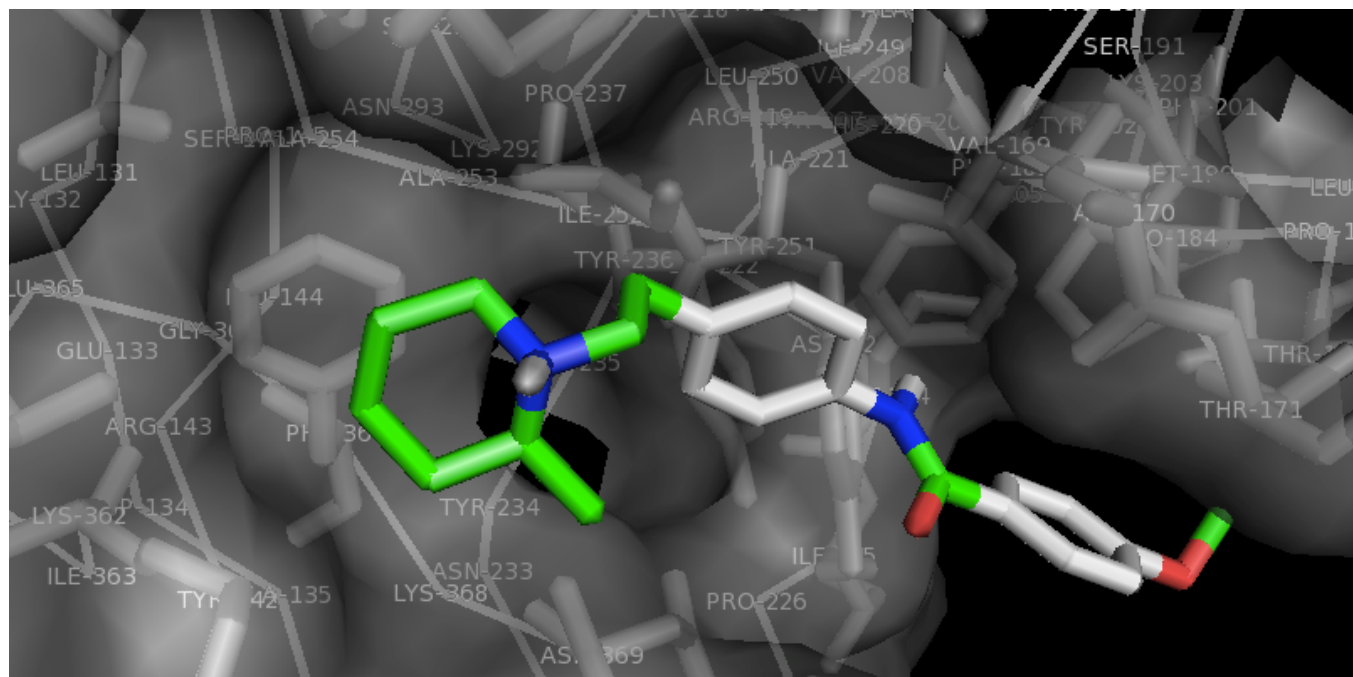

**Compound 3**  
**(ZINC3831142)**

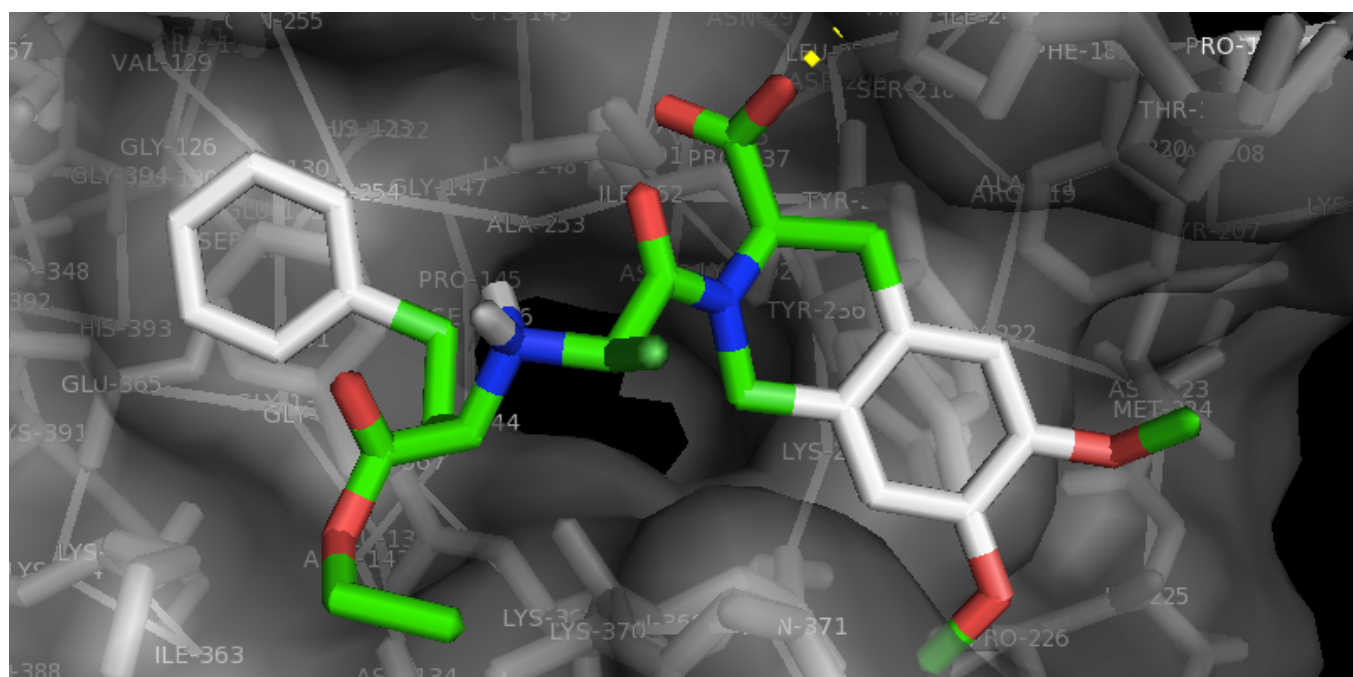

**Compound 4  
(ZINC1003)**

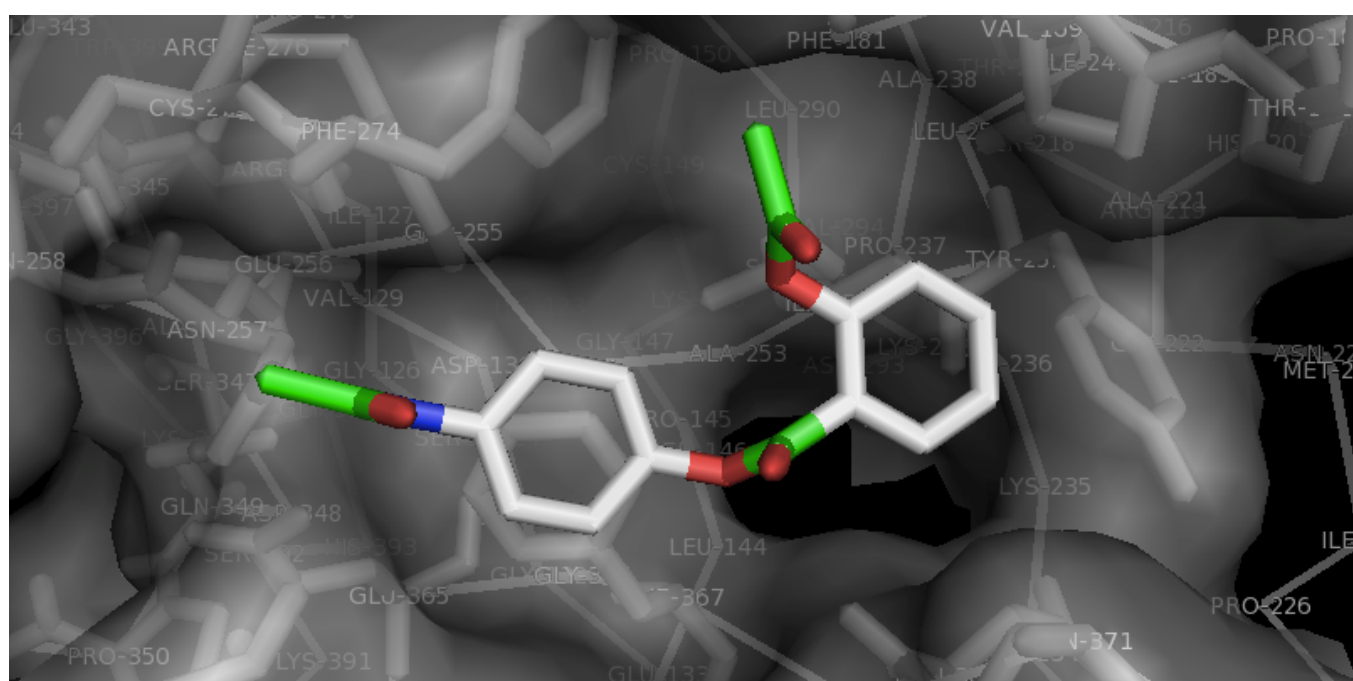

**Compound 5  
(ZINC3831140)**

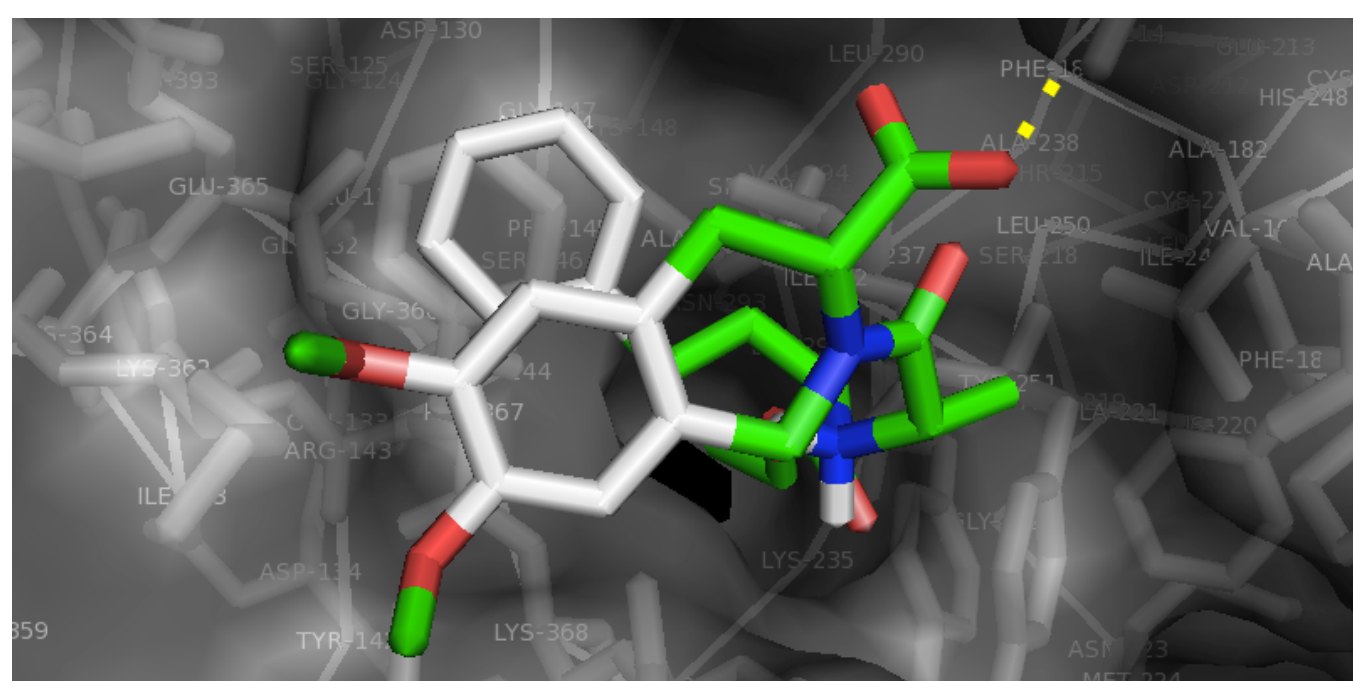

Supplement: Supplementary file 1 — Table S1: List of top 50 peptidomimetic compounds obtained by virtual screening against 6-residues (Pro-2033, Phe-2038 to Arg-2041 and Pro-2044) from PfRON2 peptide with pepMMsMIMIC server. Table S2: List of compounds targeting hydrophobic groove of PfAMA1 and having structural similarity with top 5 peptidomimetics obtained by virtual screening with pepMMsMIMIC server . Fig. S1. Zoomed view of docked structures of top 5 small drug-like molecules on the hydrophobic groove of PfAMA1. [file 642391.f1.zip › 642391.f1/Figure S1.pdf]
